# Hunger and Thirst and Some More of That Sort

**Published:** 1909-01

**Authors:** C. A. F. Lindmorme

**Affiliations:** Atlanta, Ga.


### HUNGER AND THIRST AND SOME MORE OF THAT SORT.

---

BY DR. C. A. F. LINDMORME, ATLANTA, GA.

---

I have said it at other places, and must insist upon it, that hunger and thirst, with some other notions, are not of a physical nature, but entirely psychological. In order to be corporeal they would have to be apt to show up in bodily specimens, which they do not; if they did, they would have to be presented by the investigator outwardly, and experimental proof which has never been afforded; while the proof that they are spiritual notions is being made every day, if only by the scholars the proper attention is paid to the matter; the difference is glaring enough. Hunger and thirst are an inwardness, never explored except as an inwardness. And so, gravitation, cohesion and adhesion, also pain, are spiritual notions. They have never been shown in fractions, or in portions, or regionally; gravitation, cohesion, adhesion, pain, enjoyment, are spiritual notions, a mentality; they are neither a compound nor an element; *they are ever a totality.* There is neither a greater gravitation, nor a greater appetite.

Hunger and thirst may show in extremes, but they show never by extremities, which they ought do to, however, if they were bodily items. So it is with adhesion, cohesion, gravity, pain, ect. Sir Isaac Newton concluded gravitation from the bodily movements, but never gave out gravitation as a chemical substance; he did not tell us at all what he thought it was, but left it an uncertainty the supplementation of which we can rationally only find in mentality, the mentality of nature.

According to the ordinary conception gravity has its seat in the center of the earth. This is a very incoherent and lame reasoning. It is an impracticable theory. It is altogether one sided; there is no reason to attribute the force to one part only; to make it attraction only; but why should not the same force of nature live in the meteor? a meteor, in proportion to our planet is of too trifling a size to exhibit an indepenednt gyration, and is overwhelmed by the action of the earth, and consequently it is swallowed, absorbed by the enormous swing-force of the globe; but it is doubted by the same gravitation living in the increment from outside.

It is evident that hunger and thirst are no specific bodily objects; they never existed outwardly. They are an inwardness, totally, mind, or spirit or soul, a general feeling by which alone we can perceive the soul, or other mental object. It is only figuratively that we speak of a hunger like a wolf, because usually the wolf has to wait long for his meals. But it is withal, the same predicament; the simile is indistinct; just on acocunt of the mental nature of all hunger. When a field of cauliflower has been without irrigation for a short while it exhibits in its impoverished ten times the length of endurance of drougth, we hardly notice looks the nature of its thirst. If a field of cotton is exposed to any deprivation at all, and so throughout nature the same variation; man, beast and plant. The stomach and ulterior alimentary canal of an inebriate is undergoing a desolate ruination extending even to liver and kidneys. I had a patient once, twenty years old, who drank cognac out of tumblers and by the bottle at a sitting. Should that not set the intestines on fire? Fire would have acted similarly. But yet the condition set up was not his thirst. His habit was. But then his habit was again spirituality, a compulsion which was neither cognac nor burnt up intestine, but just rack and ruin of his mind, or spirit, or soul, so that his cure was, first thing and foremost, the breaking in of abstinence. The patient I mentioned above I met once, shortly after an altercation, because his insensate drinking was threatening the house with a case of delirum tremens. He walked the streets solitarily, and I stepped up to him and invited him to take tea with be and and my wife. He accepted eagerly, and I got him to moderate himself during the rest of his term in the agricultural college which he attended.

It is the same with hunger. Leaving pronounced boulaemia out of consideration, because of its rarity, we may aver that over-eating is a very common occurence. The average man, especially the well-to-do one and mostly the rich, do not eat what they need, and would do them good, but as much as they think their stomach can bear, and moreover not what is good for them, but what they fancy and crave from habit of long standing. Virtue in our civilization is negative; the law-abiding man does not ask himself, what can I do to merit the record of a citizen, who, as much as he can, furthers the interest of the society I am living in, but shrewdly studies how must I act to evade the law, but yet pander to my greed and egoism, so the hygienic budget of the healthy man investigates how far he can go in his pleasant disdain of the rules of hygiene without incurring the risk of having to call in the M. D. Abernethy was right when he claimed, "the first disease by a debauch was made." Now, then, is the too much in the quantity only which was eaten. No, it is not; in right classification the things which tend to make up the bill of fare are bodies, subject to physical exploration, but the overeating in consequence of too much indulgence is a case of insanity, if you choose. It can not be designated differently. So is the setness of opinion that it is only meat which is a strengthening diet, and so is the uncouth obstinacy to keep at arm's length the argument that carnivorims is of closest kindred to cannibalism, a rudiment of evolution, indeed. The nitrogenous alimentation through bread out of whole wheat is fully adequate to that of the steaks and roasts, but does not pander to the long list of epidemic diseases of which the germ is harbored in the pork, beef and mutton-diet of the unphilosophical consumer.

The first rule of all hygiene is the limitation of the meals to only one course. Does it need more to condemn the banquets where there are seldom served less than half dozen? If a dish of porridge is not anything appetizing, then there is neither an occasion on hand to eat an hors d'oeuvre, and if a crust of graham bread is disdained, there is no need for a partridge either.

This seems to be all quite pedantry. It is not. I can array an experience of 75 years, and claim from the height of it, that vegetarianism exceeds carnivorism, taken all together, not only while being at table, but afterwards. With vegetarian habit there is no digestion-fever, nor is there mental irritation throughout life as in carnivorism, especially where indulgence in three times a day. There is a safe-guard in the observance of skipping meals. But if the meat-eaters think that the prescription of flesh-diet encroaches upon the enjoyment of the wonted repasts, they are very much mistaken; it is now already 28 years since I excluded meat from my diet, but I should lie if I were to admit that I do not enjoy my meals as I did formerly. If my dishes are more simple and plain, there is a compensation in the fineness of my taste. It took me half a year to establish the habit of vegetarianism, but am now so powerfully unsused to it, that I should consider it as a sacrifice to have to give it up.

If my thesis that hunger and thirst are psychological items need yet an affirmation, it must be found certainly in the argument which the taste affords. Have we not pathological conditions, to excess, indeed, where depletion exists, and yet no hunger sets in? Moreover, what of the spirituality of love—hunger?

